# Chronic prenatal corticosterone exposure alters postpartum maternal behavior and shapes sex-specific offspring outcomes

**DOI:** 10.21203/rs.3.rs-10607522/v1

**Published:** 2026-08-14

**Authors:** Carlos Rivero-Quiles, Adeline Kong, Aryan Mehra, Daljit Kaur, Mikhail Kislin, Anita E. Autry

**Affiliations:** 1. Dominick P. Purpura Department of Neuroscience, Albert Einstein College of Medicine, Bronx, NY; 2. Department of Biological Sciences, Fordham University, Bronx, NY; 3. Department of Neurobiology and Behavior, Cornell University, Ithaca, NY; 4. Department of Psychiatry and Behavioral Sciences, Albert Einstein College of Medicine, Bronx, NY

## Abstract

Stress during the prenatal period increases the risk in the next generation for negative health outcomes, including cardio-metabolic dysfunction and psychiatric diseases. To standardize prenatal exposure to stress hormone levels, we administered corticosterone (20 mg/kg s.c.) chronically (C-CORT) to pregnant mouse dams daily from gestational day 12–18. We monitored physiological, neural, and behavioral endpoints in dams and their offspring at adolescence and adulthood. Dams treated with C-CORT displayed increased anxiety-like behavior and altered maternal behavior, including increased time to retrieve pups and preference for male pup odor. These behavioral changes were accompanied by increased circulating corticosterone and increased expression of mineralocorticoid receptors in perifornical area urocortin-3-expressing neurons. In offspring, we identified age- and sex-specific differences: female adolescent mice exposed to prenatal C-CORT showed enhanced novel object recognition, while adult female offspring exposed to prenatal C-CORT showed increased immobility and reduced locomotion in an open-field arena. Adult female offspring exposed to prenatal C-CORT also lacked female-typical elevation in basal circulating corticosterone levels and showed increased hippocampal expression of corticotropin-releasing factor receptor 1 (CRFR1) in the hippocampus. Reciprocal cross-fostering normalized the effects of prenatal C-CORT exposure on adult female offspring open-field immobility, basal corticosterone, and hippocampal CRFR1 expression, but did not reverse hypoactivity. Moreover, males prenatally treated with vehicle then reared by C-CORT-treated dams exhibited greater anxiety-like behavior. These findings strengthen the idea that both fetal programming and postnatal maternal environment contribute to sex-specific neurobiological, endocrine, and behavioral outcomes in offspring.

## Introduction

Maternal stress during pregnancy is widespread, with around 70.2% of U.S. mothers reporting experiencing at least 1 stressful life event the year before their infants’ births (1). Prenatal maternal stress has been associated with increased risk of preterm birth, impaired fetal growth, and alterations in psychological and physical development of the child (2–6). Long-term health consequences of prenatal stress in offspring include increased risk of cardiometabolic outcomes, such as cardiovascular disease and diabetes (7, 8). Because prenatal stress occurs during periods of rapid fetal brain development, its effects on neural organization may contribute to persistent emotional, behavioral, and cognitive sequelae (4, 9–13).

To study the neurobiological mechanisms underlying these long-lasting effects, researchers have developed animal models of prenatal stress. Maternal gestational stressors, including chronic variable stress and restraint stress, have distinct sex- and age-dependent impacts on offspring outcomes such as hypothalamic-pituitary-adrenal (HPA) axis reactivity, immune or metabolic changes, and brain development, according to the severity of stress and the gestational window of exposure during which the stressor occurred (14–19). Despite this heterogeneity, the data together support the notion that prenatal stress affects “fetal programming” (19, 20), leading to long-lasting effects on the offspring.

The mechanisms by which maternal stress contributes to fetal programming are not well understood. A hyporesponsive HPA axis buffers maternal stress responses during pregnancy, and fetal exposure to maternal circulating glucocorticoids (cortisol in humans, corticosterone in rodents) is limited by placental expression of 11β-hydroxysteroid dehydrogenase type 2 (Hsd11b2), an enzyme that converts maternal glucocorticoids into inactive forms (21, 22). Emerging evidence suggests that maternal glucocorticoids impact fetal development by altering gene expression in the placenta through suppression of Hsd11b2 activity, thereby allowing maternal glucocorticoids to reach the fetus, or through other genetic reprogramming of the placenta that affects fetal growth and development (19, 21, 23–27). Whether the impact of maternal glucocorticoids on the fetus is direct or indirect, these studies agree that maternal glucocorticoids play a critical role in the persistent effects of prenatal stress on offspring. While prior research has predominantly focused on studying fetal reprogramming that occurs at the time of stress, evidence shows that prenatal stress also induces long-lasting changes in dam physiology and behavior (28–32). Altered maternal caregiving behavior, cognition, and stress responsivity after prenatal stress affects the postnatal environment in which the offspring are nursed and cared for (30–33).

In the present study, we aimed to test the hypothesis that long-lasting behavioral and neurobiological changes in offspring prenatally exposed to elevated maternal glucocorticoids are mediated by maternal caregiving. We used chronic corticosterone (C-CORT) administration in pregnant mice to gain experimental control over the level of elevated maternal glucocorticoids to which the fetus was exposed (34). We focused on the last week of gestation (GD12–18) to capture the sensitive period for fetal hippocampal and cortical neurogenesis (35–38). We find that prenatal C-CORT treatment affects maternal glucocorticoid secretion, maternal exploratory behavior, appetitive maternal caregiving, and expression of mineralocorticoid receptors in the maternal hypothalamus. In offspring, we observe age- and sex-specific effects of prenatal C-CORT treatment such that females show enhanced novel object recognition in adolescence and increased immobility in the open field test in adulthood. This change in adult female behavior was associated with blunted corticosterone secretion and increased hippocampal expression of corticotropin-releasing factor receptor 1 (CRFR1). At the time points studied, male offspring were unaffected. Cross-fostering normalized immobility behavior of adult female offspring exposed to prenatal C-CORT and rescued circulating corticosterone levels and expression of hippocampal CRFR1. Taken together, these results confirm that prenatal exposure to excessive maternal corticosterone leads to long-lasting behavioral changes in both the dam and offspring, and certain sex-specific effects of prenatal C-CORT exposure on neurobiology, physiology, and behavior of adult offspring are moderated by maternal care.

## Materials and Methods

### Animals

Pregnant female (GD4) C57BL/6J mice, aged 8–10 weeks, were ordered from Jackson Laboratory (Bar Harbor, ME), were singly housed, and habituated to our facility for 7 days before experimental manipulation. Mice were maintained in a temperature- and humidity-controlled room on a 12h:12h dark light cycle (10:30 am- 10:30 pm dark phase) with access to food and water ad libitum. Pups remained with the dam through weaning. Dams were randomly allocated to experimental groups. All experiments were performed according to the ARRIVE ethical guidelines and NIH guidelines and approved by the Albert Einstein College of Medicine Institutional Animal Care and Use Committee (protocol 20180110; 20180111; 00001386). Dams were weighed every other day. No pups were culled in this study. Offspring were weaned from the experimental dams at postnatal day 21 (PND 21) and separated by sex. Offspring were randomly assigned to adolescent or adult groups for further testing (Fig. 1A).

### Prenatal Corticosterone Administration

Corticosterone (CORT; Sigma-Aldrich, St. Louis, MO, USA) was dissolved in 5% ethanol in sesame oil (Sigma-Aldrich, St. Louis, MO, USA) and administered at a dose of 20 mg/kg subcutaneously (s.c.) to the nape of the neck. Vehicle mice were injected with sesame oil at the same volume. CORT dose was chosen based on our prior study (34). Injections began on gestation day 12 (GD12) and ended on GD18, for a total of 7 injections (Fig. 1A). Injections were administered within 2 hours of the dark phase onset.

### Cross-fostering

Dams were treated with corticosterone or vehicle as described above. Within 24 hours of birth, pups were swapped between a C-CORT and vehicle dam with equivalent litter sizes.

### Serum Corticosterone Measurement

Serum was isolated from whole blood collected on the day of sacrifice. Corticosterone was measured by EIA (IDS, Fountain Hills, AZ, USA) as previously described (34). For complete details, see **Supplemental Methods**.

### Behavior Assays

Experiments were conducted during the dark phase under dim red light. Tests were recorded by Fly Capture cameras (Point Grey, Richmond, BC, Canada), and behaviors were scored by an observer blinded to experimental conditions using Ethovision XT 13 or 18 (Noldus Information Technology, Leesburg, VA, USA).

#### Appetitive Maternal Behavior

Appetitive maternal behavior tests were conducted in the mouse’s home cage as previously described on postnatal day 10 (PND 10; Fig. 1A) (34, 39–42). For complete details, see **Supplemental Methods**.

#### Behavioral sequences analysis and Transition Probability Analysis

Behavioral sequences analysis and Transition Probability Analysis were conducted as previously described (34). For complete details, see **Supplemental Methods**.

#### Open-field exploration

Mice were assessed for activity in a 45 cm × 45 cm open-field at 40 lux for 5 min as previously described (43). See **Supplemental Methods** for more details.

#### Odor preference test

Odor preference was assessed in the open-field arena, adapted from Zou et al. 2015 (44). See **Supplemental Methods** for more information.

#### Elevated plus maze

The elevated plus maze was conducted as previously described (45). See **Supplemental Methods** for more details.

#### Novel object recognition test

Novel object recognition training and testing were conducted in the open-field arena as previously described (46). See **Supplemental Methods** for more information.

### Fluorescence In Situ Hybridization

Fluorescence in situ hybridization (FISH) was performed as recommended by ACD Bio (Newark, CA, USA) using V2 RNAscope reagents. Ucn3 (Cat No. 464861), MR (Nr3c2; Cat No. 456331), GR (Nr3c1; Cat No. 475261), and CRFR1 (Cat No. 418011) probes were used per manufacturer’s instructions. For further details, see **Supplemental Methods**.

### Image Analysis

Images were exported from Zen Blue software after linear spectral unmixing. For maternal brains, colocalization analysis was conducted as previously described (34), see **Supplemental Methods** for more details. For offspring brains, mean fluorescence intensity was measured in the pyramidal cell layers of dentate gyrus (DG), CA1, and CA3 for each channel in FIJI (ImageJ, NIH). For further details, see **Supplemental Methods**.

### Data Analysis and Statistics

Data were analyzed in Graphpad Prism 10 with the investigator blinded to experimental condition. Sample sizes were selected based on previous experiments (34, 39, 41–43). See **Supplemental Methods** for further details. Outliers were identified by Grubb’s outlier test or ROUT. P values reported as follows: * P<0.05, ** P<0.01, *** P<0.001, **** P<0.0001. All data are expressed as mean ± SEM.

## Results

### Chronic prenatal corticosterone has long-lasting effects on dam hormone secretion and exploratory behavior

In our initial experiments, we treated pregnant C57BL/6J mice with chronic corticosterone (C-CORT, 20mg/kg) (34) or vehicle (sesame oil) from GD12-GD18. We examined appetitive maternal behavior, odor preference, and open-field exploration in dams; then, in separate cohorts, we examined offspring behaviors at both adolescent and adult time points (Fig. 1A, top). In dams, we tested open-field behavior on PND9, appetitive maternal behavior on PND10, odor preference on PND18, then after pup weaning (PND21), we sacrificed the dams (PND22) to collect brains and blood (Fig. 1A, bottom). Throughout the experiment, we monitored body weight, food intake, and fluid intake of the dams, and these measures were indistinguishable between groups (Fig. 1B–D). We found that C-CORT-treated dams spent less time in the center (Fig. 1E) and more time in the border (Fig. 1F) of an open-field arena. This group difference was not attributable to changes in distance moved, velocity, or immobility in the open field (Fig. 1G–I). In a pilot study, we collected dam serum at GD18 and observed a significant reduction in circulating corticosterone in C-CORT-treated dams (Fig. 1J), consistent with our previous study (34). However, at PND22, we observed a significant increase in circulating corticosterone in C-CORT-treated dams (Fig. 1K). Altogether, these results suggest that chronic prenatal corticosterone treatment has a long-lasting effect on regulation of HPA axis responses and anxiety-like behavior without affecting fluid balance, food consumption, or overall activity levels.

### Chronic prenatal corticosterone impacts appetitive maternal behavior and dam preference for male pup odor

On PND10, we examined appetitive maternal care by placing 2 newborn (P0-P1) pups from a different dam into the experimental dam’s undisturbed home cage (Fig. 1A, Methods). We quantified behaviors for 10 minutes following the initial investigation of the pups (Supp. Fig. 1A). Dams treated with prenatal C-CORT spent significantly more time retrieving pups compared to vehicle-treated dams (Fig. 2A). However, latency to retrieve the 1^st^ and 2^nd^ pup was similar between groups (Supp. Fig. 1B–C), consistent with our observation that C-CORT-treated dams make more unsuccessful retrieval attempts than vehicle-treated dams (vehicle retrievals median 2, C-CORT retrievals median 3, **p<0.0078 Wilcoxon signed rank two-tailed t-test). We also found that prenatal C-CORT-treated dams spent significantly more time investigating the pups and trended toward increased time spent grooming them (Fig. 2B–C). No group differences were observed in the time spent in the nest (Fig. 2D), crouching over the pups, or rearing (Supp. Fig. 1D–E).

We next studied behavioral syntax shift, switching rate, LZC, and transition entropy, to study the impact of prenatal C-CORT on caregiving patterns. Overall, we identified trends toward increased probabilities of shifts between crouching and pup grooming and between spending time in the nest and retrieving (Supp. Fig. 1F). Both groups of dams showed significantly reduced switching rates (Fig. 2E) and LZC normalized (Fig. 2F) during the caregiving phase (time after retrieval of the 2^nd^ pup marked by black bar in Supp. Fig. 1A). This outcome is consistent with our previous study illustrating that the retrieval phase is characterized by flexible, dynamic behavior sequences while the caregiving phase consists of more organized, stereotyped behavior patterns (34). Behavior transition entropy was not affected by prenatal C-CORT treatment (Fig. 2G). Overall, these results suggest that prenatal C-CORT treatment affects appetitive maternal caregiving behaviors that primarily occur during the retrieval phase.

To assess sex-specific pup-directed behavior, we conducted an odor preference test in which a dam was simultaneously presented with 2 cotton swabs containing scents from foreign wild-type pups, one with male odor, one with female odor, in an open field arena (Fig. 2H). We found that vehicle-treated dams spent similar duration in contact with both odors, while the C-CORT-treated dams spent significantly more time in contact with the male pup odor compared to the female pup odor (Fig. 2I). This difference in contact time was not attributable to differences in distance moved in the arena (Fig. 2J). This result suggests that effects of C-CORT on dam retrieval may be sex-specific, such that male pups are cared for preferentially.

### Chronic prenatal corticosterone treatment leads to lasting increases in the expression of corticosteroid receptors in hypothalamic Ucn3 neurons

Our previous studies suggested that activity of perifornical area (PeFA) Ucn3 neurons in dams is impacted by perinatal stressors (34, 40). Here, we examined glucocorticoid receptor (GR; NR3C1) and mineralocorticoid receptor (MR; NR3C2) expression in dams with prenatal vehicle or C-CORT treatment at PND22 (Fig. 2K–L). We observed that prenatal C-CORT treatment significantly increased MR expression in the PeFA Ucn3 neurons (Fig. 2M–N). We also observed a trend toward increased GR expression in Ucn3 neurons (Fig. 2O–P). Together, these results show that PeFA Ucn3 neurons express corticosteroid receptors and adaptively respond to prenatal C-CORT treatment in dams by persistently increasing MR expression.

### Adolescent female offspring of C-CORT-treated dams show enhanced novel object recognition

To study the impact of prenatal C-CORT on the next generation, we examined behavior and circulating corticosterone levels in male and female offspring at both adolescent and adult life stages in separate cohorts. In the adolescent cohorts, we studied open-field behavior at PND33, conducted novel object training and testing on PND34–35, and collected trunk blood at PND36 (Fig. 3A). Male and female offspring from both treatment groups showed similar exploratory behaviors in the open-field arena (Fig. 3B–F). In the novel object recognition task, very few mice showed a preference for the novel object, suggesting that the 24-hour interval between training and testing presented a challenging task for adolescents (Fig. 3G). However, we observed increased investigation of the novel object in the female adolescent offspring of C-CORT-treated dams (Fig. 3G). Together, these results suggest that prenatal C-CORT-treatment affects cognitive abilities in adolescence in a sex-specific manner.

### Adult female offspring of C-CORT-treated dams show reduced exploratory behavior

In a separate offspring cohort, we studied the same behaviors in adulthood (Fig. 3H). We noticed a trend toward decreased exploration of the center of the open-field in adult female offspring of C-CORT-treated dams (Fig. 3I), with no changes in border exploration among groups (Fig. EJ). We also observed increased immobility in adult female C-CORT offspring (Fig. 3K) accompanied by reduced distance moved (Fig. 3L) and velocity (Fig. 3M). Together, these results suggest that adult female C-CORT offspring show depressive-like behavior in the open field.

In adults, most mice showed a preference for the novel object (Fig. 3N), revealing that the 24-hour testing time point was easier for adult mice to learn compared to adolescents. In adults, discrimination of the familiar vs. novel object was similar across both treatment groups and sexes (Fig. 3N). These results show that the effect of prenatal C-CORT on cognitive abilities is both sex- and life-stage-specific.

### Cross-fostering normalizes the impact of prenatal C-CORT on adult female offspring immobility in the open field

To address whether the behavioral impacts of prenatal C-CORT in offspring were related to in utero C-CORT exposure or to subsequent maternal care, we performed a cross-fostering experiment in which pups from vehicle- or C-CORT-treated dams were swapped to a dam of the opposite treatment group within 24 hours of birth (Fig. 4A). We then examined behavior and collected brains and trunk blood from adult offspring of both sexes. In cross-fostered adults, we observed indistinguishable time spent in the center of the open-field (Fig. 4B), but significantly increased time spent in the border in adult male offspring cross-fostered from vehicle→C-CORT dams compared to female littermates (Fig. 4C). We noted that cross-fostering normalized group differences in immobility in the adult female offspring (Fig. 4D). However, both groups of females showed increased immobility compared to their male littermates (Fig. 4D). With respect to velocity in the open-field, we observed that adult female offspring cross-fostered from C-CORT→vehicle dams were significantly slower compared to their male littermates (Fig. 4E–F). Adult female offspring cross-fostered from C-CORT→vehicle dams also showed significantly reduced distance traveled compared to both male littermates and females cross-fostered from vehicle→C-CORT dams.

In novel object recognition behavior, we identified no sex- or treatment-based differences (Fig. 4G). To further examine exploratory behaviors, we tested cross-fostered adults in the elevated plus maze. We found no group/sex differences in time exploring the open arms (Fig. 4H). We observed increased time spent in the closed arms in male offspring cross-fostered from vehicle→C-CORT dams compared to female littermates (Fig. 4I), but no changes in immobility among group/sex in this assay (Fig. 4J).

Taken all together, important interactions among prenatal C-CORT exposure, maternal care, and offspring sex begin to emerge. We find that cross-fostering normalized immobility differences in adult female offspring exposed to C-CORT prenatally, suggesting that maternal care may more strongly affect adult depression-like behavior relative to prenatal C-CORT exposure. We additionally observe that male offspring cross-fostered from vehicle→C-CORT dams show increased anxiogenic behavior in both the open field and elevated plus maze, suggesting that maternal care from C-CORT-treated dams leads to sex-specific effects in anxiety-related behavior. These results together reinforce the idea that maternal care in the postnatal period can have long-lasting sex-specific effects on offspring. Importantly, however, not all effects of prenatal C-CORT treatment were reversed. Adult female offspring transferred from C-CORT-treated to vehicle-treated dams continued to exhibit reduced velocity and distance traveled in the open field (Fig. 4E–F), suggesting that in utero C-CORT exposure and postnatal maternal care exert distinct, sex-specific effects on exploratory behavior in adulthood.

### Cross-fostering normalizes sex-differences in circulating corticosterone and hippocampal CRFR1 expression

To study the impact of cross-fostering on neurobiology and physiology, we examined circulating corticosterone and hippocampal expression of corticotropin-releasing factor receptor 1 (CRFR1). We did not observe differences among sex/group in circulating corticosterone in adolescent offspring (Fig. 5A). In adult offspring, we observed a significant increase in circulating corticosterone in adult females with prenatal vehicle treatment compared to male littermates (Fig. 5B), consistent with previous studies showing sex differences in circulating corticosterone in rodents under baseline conditions (47–49). However, this sex difference was blunted in adult offspring with prenatal C-CORT exposure. Cross-fostering normalized the differences in adult female circulating corticosterone levels (Fig. 5C), suggesting that maternal care from a C-CORT-treated dam suppresses the sex-specific increase in circulating corticosterone in adult females born to vehicle-treated dams.

We next examined the expression of CRFR1 in the dorsal hippocampus of male and female adult offspring from adult (Fig. 5D) and cross-fostered (Fig. 5E) cohorts. We focused on the hippocampus because of its well-established roles in learning and memory, stress regulation, and affective behaviors (50–53), behavioral domains that were impacted in our previous experiments. We focused on CRFR1 as a potential mediator of HPA axis feedback on the hippocampus, including in adults after prenatal stress exposure (54–57). We observed a striking sex difference in the offspring of C-CORT-treated dams, with CRFR1 expression significantly upregulated in females compared to males in subregions of the dorsal hippocampus, particularly the CA1 (Fig. 5F). This result suggests that the observed sex differences in open-field exploratory behavior could be mediated by hippocampal changes in CRFR1 expression.

We next examined how cross-fostering affected hippocampal CRFR1 expression. We discovered that cross-fostering normalized sex differences in CRFR1 across hippocampal subfields, including the CA1 (Fig. 5G). This result suggests that the behavioral impact of cross-fostering may be in part attributable to normalization of hippocampal CRFR1 expression among sex/treatment groups.

## Discussion

Here, we show that chronic corticosterone treatment in mid-to-late gestation produces persistent changes that impact physiology, neurobiology, and behavior in both dams and their offspring from adolescence to adulthood. In dams, we observed that prenatal C-CORT led to anxiogenic behavior, increased appetitive behavior toward pups, and biased the dam toward investigating male pup odor weeks after termination of C-CORT treatment. These behavioral changes were accompanied by long-lasting elevation of maternal corticosterone levels and increased mineralocorticoid receptor expression in PeFA Ucn3 neurons.

In offspring, we observed sex- and age-dependent changes in behavior and physiology. Female offspring of C-CORT-treated dams showed enhanced novel object recognition in adolescence, while adult female offspring showed increased immobility and reduced locomotion speed and distance in the open field arena, suggestive of a depression-like phenotype. These behavioral changes suggest that C-CORT treatment prenatally affects the trajectory of brain development specifically in female offspring, such that brain areas related to cognition develop more rapidly compared to males, whereas brain areas regulating depression-related behavior may develop abnormally between adolescence and adulthood. This altered development in females could be attributed to changes in dam caregiving, given that dams with prenatal C-CORT treatment prefer male pup odor and thus may spend more time investigating and retrieving male pups. Additionally, females exposed to C-CORT prenatally did not show a female-typical elevation of corticosterone in adulthood, which could also influence depression-like behavior.

### Maternal care as a critical mediator of prenatal stress on specific offspring outcomes

Our cross-fostering results indicate that maternal care critically mediates the impact of prenatal exposure to C-CORT on behavior, circulating stress hormone, and hippocampal expression of CRFR1. This work fits into a larger body of work showing that gestational stress alters maternal caregiving in rodents, which can persist through subsequent litters (28, 33, 58–60). Studies have also shown that cross-fostering can overcome prenatal stress-induced behavioral changes in offspring, whether behavior differences were related to pre-conception (61) or gestational stress (62, 63). In our study, we identified a potential mechanism for caregiving impacts on sex-specific behavior via increased preference for male pups in C-CORT-treated dams. A prior study showed that mouse dams experience enhanced odor memory, which is compromised by gestational restraint stress potentially through altered olfactory bulb neurogenesis (64). Our current results extend this growing body of work to include prenatal exposure to excess corticosterone in mice.

### Limitations of the current study and future directions

We used cross-fostering to distinguish fetal programming versus postnatal/maternal environment effects of prenatal C-CORT treatment. However, we did not include a control in which offspring were cross-fostered within the same maternal treatment groups, nor did we measure maternal caregiving in the cross-fostering experiment. A recent study demonstrated that cross-fostering can lead to differences in maternal care toward the pups (58, 63), so assessment of caregiving in the presence of cross-fostering is a key outcome measure for future studies.

Several groups have used a similar prenatal corticosterone treatment to assess sex-specific effects on offspring outcomes, including adrenal development (65), allergic airway inflammation (66), hypotension (67), and renal adaptations (68). One study suggests that prenatal corticosterone exposure alters placental gene expression in a sex-specific manner (69), highlighting a potential mechanism for prenatal C-CORT to influence fetal programming. It would be interesting to examine placental and other offspring health outcomes in the context of cross-fostering to tease apart the extent to which these disparate impacts are mediated by fetal programming, postnatal environment, or interactions between them.

In the maternal C-CORT treatment group, we also did not assess how much corticosterone may have been passed from the dam to the offspring during the postnatal period. Prior studies have shown that prenatally stressed dams do not show differences in milk corticosterone levels at PND9, but this remains to be tested with prenatal C-CORT treatment, particularly in light of the observed long-lasting changes in maternal corticosterone (Fig. 1K).

Finally, in the present study, we did not perform a mechanistic assessment of either how MR expression in the hypothalamus may affect maternal caregiving or how CRFR1 expression in the hippocampus mediates behavioral effects of prenatal exposure to C-CORT. A recent study highlighted the altered function of CRF pathways from the amygdala to the nucleus accumbens in mediating sex-specific effects of early life adversity, which fits with our observation of altered CRF receptor expression (70). Other prior studies have also illuminated other potential molecular targets of prenatal stress, such as epigenetic mechanisms in the hippocampus (71–74) as well as other brain areas including the cortex and amygdala (62, 75, 76), suggesting that future research on the prenatal C-CORT model could take an unbiased approach to identify additional underlying molecular mechanisms.

### Conclusions

Our work demonstrates that both fetal programming and postnatal maternal environment contribute to neurobiological, endocrine, and behavioral sequelae in offspring. While placental changes may directly mediate some sex-specific changes, we illuminate the need to consider how postnatal caregiving may contribute to or exacerbate impacts on offspring.

## Supplementary Material

1

Supplementary Files

This is a list of supplementary files associated with this preprint. Click to download.

- RiveroKongSupplementalMethodsNPPSubmission.pdf

## Figures and Tables

**Figure 1.**
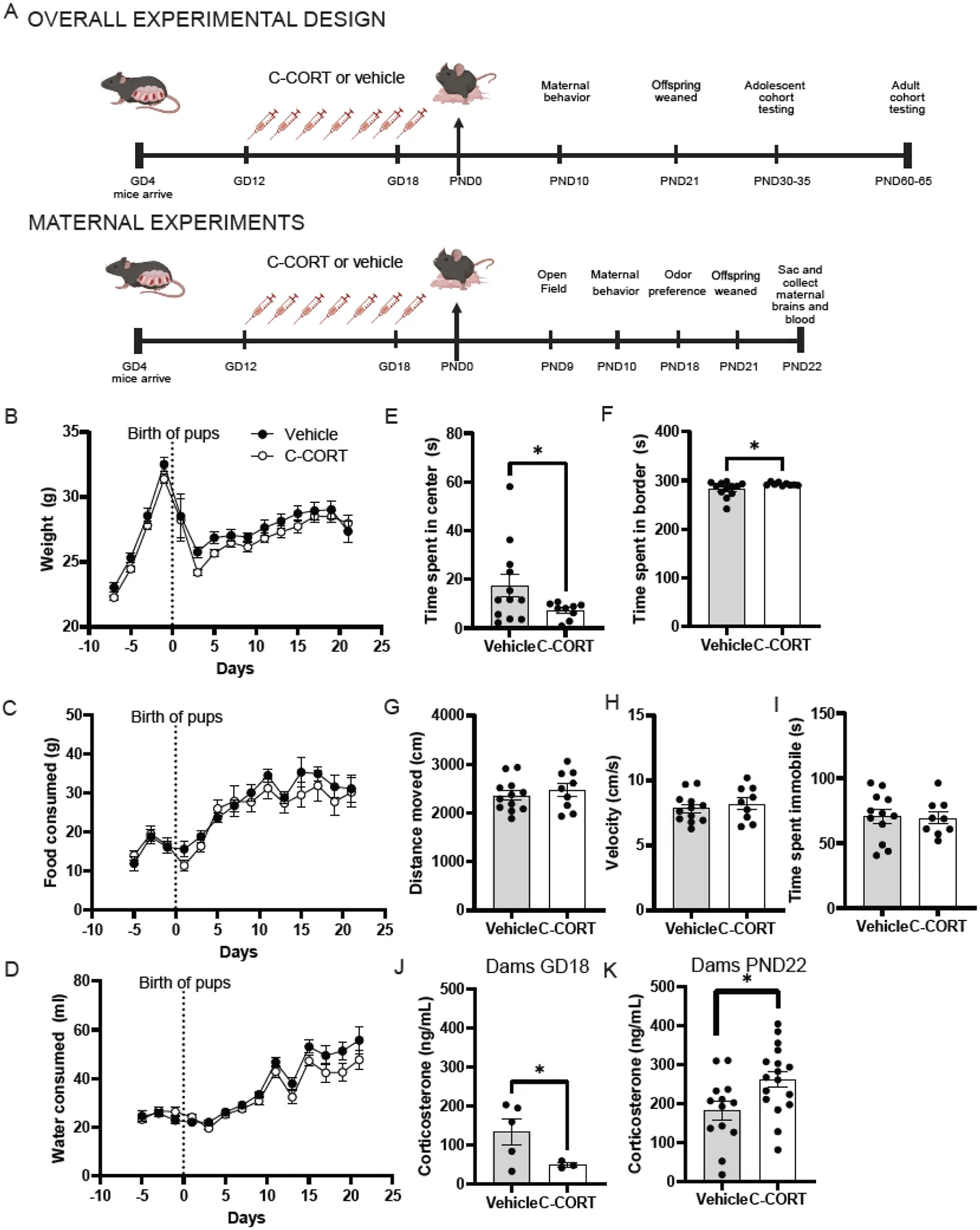
Chronic prenatal corticosterone affects exploratory behavior and circulating corticosterone in dams. A. Overall behavior paradigm. B. The weight of dams throughout the experiment is not affected by C-CORT treatment. C. Food consumed by dams throughout the experiment is not changed with C-CORT treatment. D. Water consumption is not affected by C-CORT treatment. E. Time spent in the center of an open field arena is reduced in dams treated with C-CORT (Mann-Whitney test, one-tailed, U=29, *p=0.0393). F. Time spent in the border of an open field arena is increased with C-CORT treatment (Mann-Whitney test, one-tailed, U=28, *p=0.0333). G. Distance moved, H. Velocity, and I. Immobility in the open field arena is comparable between vehicle and C-CORT-treated dams. J. Circulating corticosterone decreased at the end of the treatment course on GD18 in C-CORT-treated dams (Welch’s t-test, one-tailed, t=2.550, df=4.199, *p=0.0302). K. Circulating corticosterone increased at the time of pup weaning on PND 21 in C-CORT-treated dams (Unpaired t-test, two-tailed, t=2.469, df=28, *p=0.0199).

**Figure 2.**
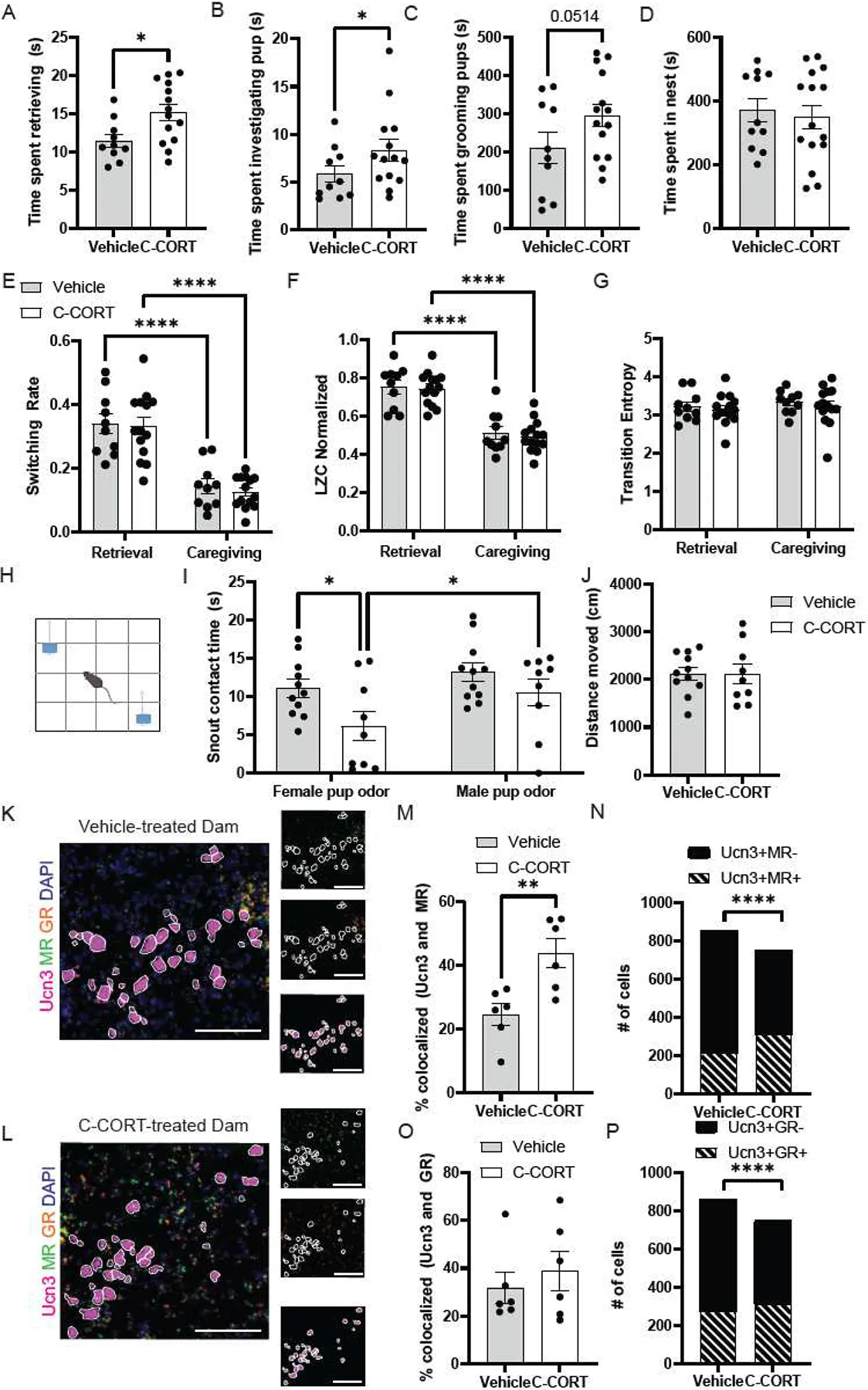
Maternal care at PND10 is affected by prenatal C-CORT treatment. A. Time spent retrieving is significantly increased in C-CORT-treated dams (Welch’s two-tailed t-test *p=0.0117, t=2.748, df=21.99). B. Time spent investigating pups is significantly increased in C-CORT-treated dams (Welch’s one-tailed t-test *p=0.0461, t=1.761, df=21.90). C. Time spent grooming pups trends toward an increase in C-CORT-treated dams (Welch’s one-tailed t-test p=0.0514, t=1.719, df=17.95). D. We observed no group differences in time spent in the nest. E. We observed a significantly higher switching rate in dams of both groups during the retrieval phase (Two-way repeated measures ANOVA main effect of behavior phase F_1,22_=74.50 ****p<0.0001, Fisher’s post hoc test significant between retrieval and caregiving for vehicle ****p<0.0001 and C-CORT-treated ****p<0.0001 dams). F. Normalized LZC revealed significantly higher behavioral complexity in dams of both groups during the retrieval phase (Two-way repeated measures ANOVA main effect of behavior phase F_1,22_=57.18 ****p<0.0001, Fisher’s post hoc test significant between retrieval and caregiving for vehicle ****p<0.0001 and C-CORT-treated ****p<0.0001 dams). G. There were no significant differences in transition entropy between vehicle and C-CORT treated groups. H. Schematic of pup odor preference test. I. Snout contact time with female pup odor swab is reduced in C-CORT-treated dams compared both to C-CORT treated dam contact time with the male pup odor swab and vehicle-treated dam contact time with the female pup odor swab (Two-way repeated measures ANOVA main effect of group F_1,18_=4.563 *p=0.0467, main effect of odor F_1,18_=7.724, *p=0.0124, Fisher’s post-hoc comparisons significant between vehicle and C-CORT female odor *p=0.0273 and C-CORT male vs. female pup odor *p=0.0220). J. Distance moved in the odor preference test were comparable between groups. Representative images of fluorescence in situ hybridization in the perifornical area for Ucn3 (magenta), MR (green), and GR (orange) (DAPI, blue; scale bar 100 μm) in K. vehicle-treated or L. C-CORT-treated dams. M. Percent colocalized Ucn3 and MR cells is increased with prenatal C-CORT (Welch’s two-tailed t-test t=3.363, df=9.250, **p=0.0080). N. Proportional percentage of Ucn3+/MR+ neurons is increased in C-CORT-treated dams compared to vehicle-treated dams (two-sided Fisher’s exact test: vehicle n = 854 cells; C-CORT n = 749 cells; ****p = 0.0001; represented in bars). O. Percent colocalized Ucn3 and GR cells is not significantly different between groups. P. Proportional percentage of Ucn3+/GR+ neurons is increased in C-CORT-treated dams compared to vehicle-treated dams (two-sided Fisher’s exact test: vehicle n = 859 cells; C-CORT n = 748 cells; ****p = 0.0001; represented in bars).

**Figure 3.**
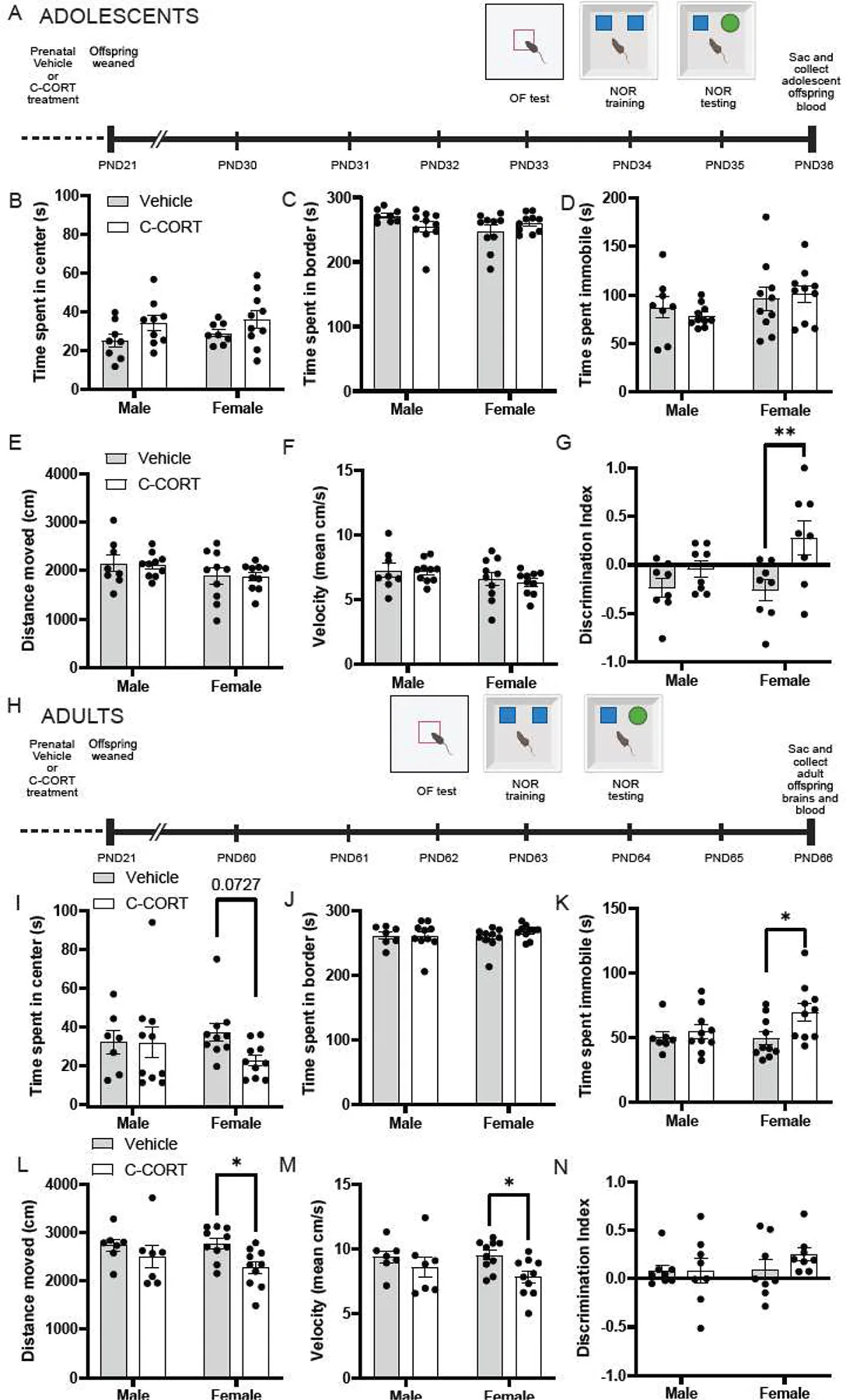
Prenatal C-CORT treatment impacts female offspring behavior differentially in adolescence versus adulthood. A. Schematic of adolescent offspring behavior testing. B. Adolescent offspring exploration of the center and C. border of an open field is similar among groups. D. Immobility, E. distance moved, and F. velocity in the open field arena are comparable among groups. G. Discrimination of a novel object is increased in female offspring of prenatal C-CORT-treated dams (Two-way ANOVA main effect of treatment F_1,28_=9.495, **p=0.0046, Fisher’s post hoc test significant between female offspring vehicle vs. C-CORT **p=0.0034). H. Schematic of adult offspring behavior testing. I. Time spent in the center of an open field arena trends toward a decrease in adult female offspring with prenatal C-CORT treatment (Two-way ANOVA Fisher’s post-hoc test between female adult offspring vehicle vs. C-CORT p=0.0727). J. Time spent in the border of an open field is comparable among groups. K. Immobility in the open field arena is increased in adult female offspring with prenatal C-CORT treatment (Two-way ANOVA significant main effect of treatment F_1,33_=4.599, *p=0.0395, Fisher’s post-hoc test significant between female adult offspring vehicle vs. C-CORT *p=0.0131). L. Velocity in the open field arena is reduced in adult female offspring with prenatal C-CORT treatment (Two-way ANOVA significant main effect of treatment F_1,30_=5.650, *p=0.0240, Fisher’s post-hoc test significant between female adult offspring vehicle vs. C-CORT *p=0.0170). M. Distance moved in the open field arena is reduced in adult female offspring with prenatal C-CORT treatment (Two-way ANOVA significant main effect of treatment F_1,30_=6.191, *p=0.0186, Fisher’s post-hoc test significant between female adult offspring vehicle vs. C-CORT *p=0.0129). N. Discrimination of a novel object is comparable among treatment groups.

**Figure 4.**
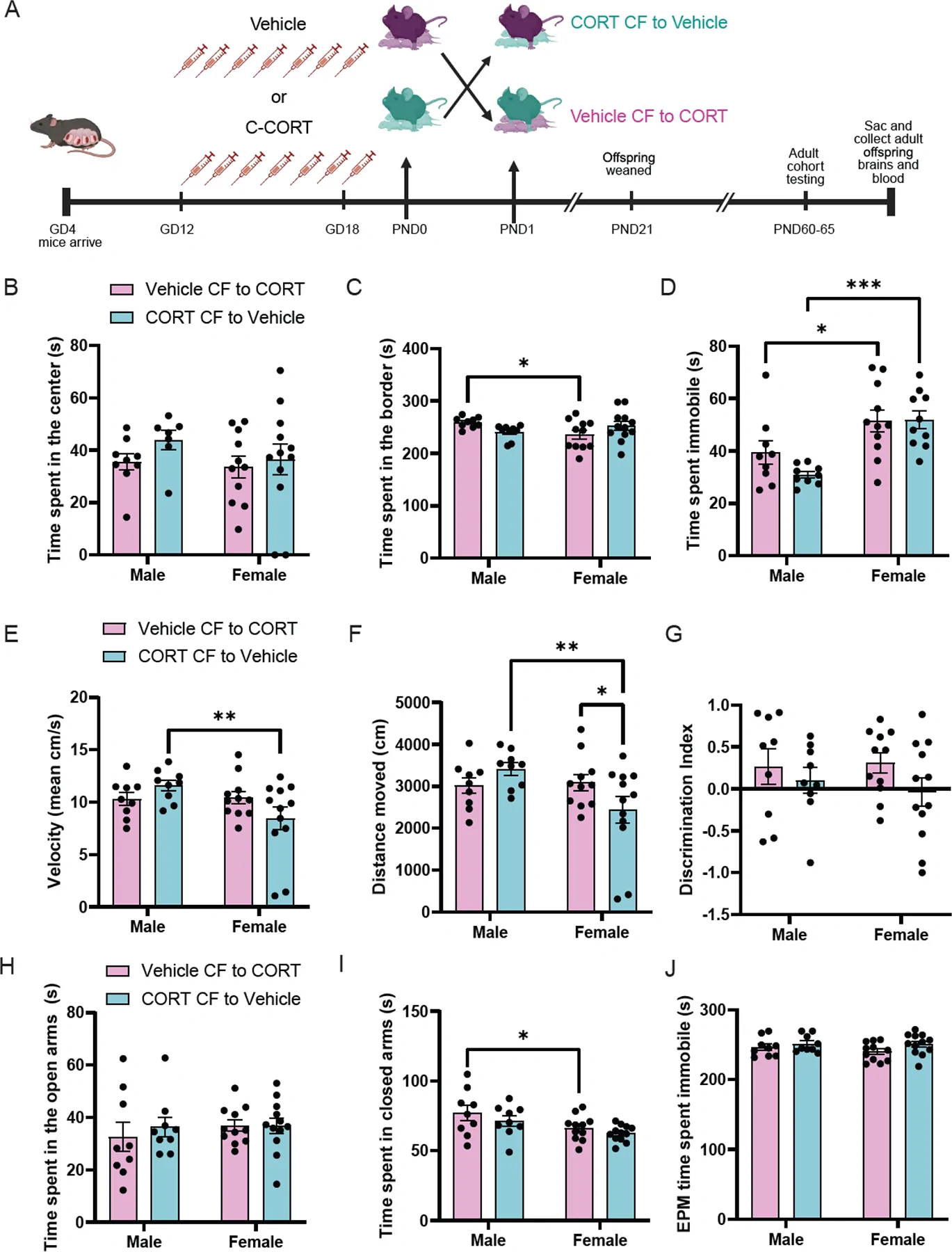
Cross-fostering offspring with prenatal C-CORT treatment normalizes open field immobility behavior in adult females. A. Schematic of cross-fostering experiment. B. Time spent in the center of an open field arena is similar among groups. C. Time spent in the border of an open field arena is increased in male offspring of prenatal vehicle-treated dams cross-fostered to C-CORT dams compared to their female offspring counterparts (Two-way ANOVA main effect of sex × treatment interaction F_1–37_=6.007, *p=0.0191, Fisher’s post-hoc test significant between male and female vehicle-to-C-CORT cross-fostered offspring *p=0.0276). D. Time spent immobile in the open field was increased in female offspring compared to male offspring from both groups (Two-way ANOVA significant main effect of sex F_1,35_=20.55, ****p=0.0001, Fisher’s post-hoc test significant between male vs. female vehicle-to-C-CORT offspring *p=0.0229 and male vs. female C-CORT-to-vehicle offspring ***p=0.0003). E. Velocity is reduced in female offspring cross-fostered from C-CORT-treated dams to vehicle-treated dams (Two-way ANOVA significant main effect of sex × treatment interaction F_1,37_=4.232, *p=0.0468, Fisher’s post-hoc test significant between male vs. female offspring of C-CORT-to-vehicle dams **p=0.0076). F. Distance moved in the open field is reduced in female offspring cross-fostered from C-CORT-to-vehicle dams (Two-way ANOVA significant main effect of sex × treatment interaction F_1,37_=4.825, *p=0.0344, Fisher’s post-hoc test significant between male vs. female offspring of C-CORT-to-vehicle dams **p=0.0058 and between female cross-fostered groups *p=0.0461). G. Novel object discrimination is not distinguishable among cross-fostered groups. H. Time spent in the open arms of the elevated plus maze is not different among groups. I. Time spent in the closed arms of the elevated plus maze is significantly increased in male offspring of prenatal vehicle-treated dams cross-fostered to C-CORT dams compared to their female offspring counterparts (Two-way ANOVA main effect of sex F_1–37_=8.228, **p=0.0068, Fisher’s post-hoc test significant between male and female vehicle-to-C-CORT cross-fostered offspring *p=0.0362). J. Elevated plus maze immobility is comparable among groups.

**Figure 5.**
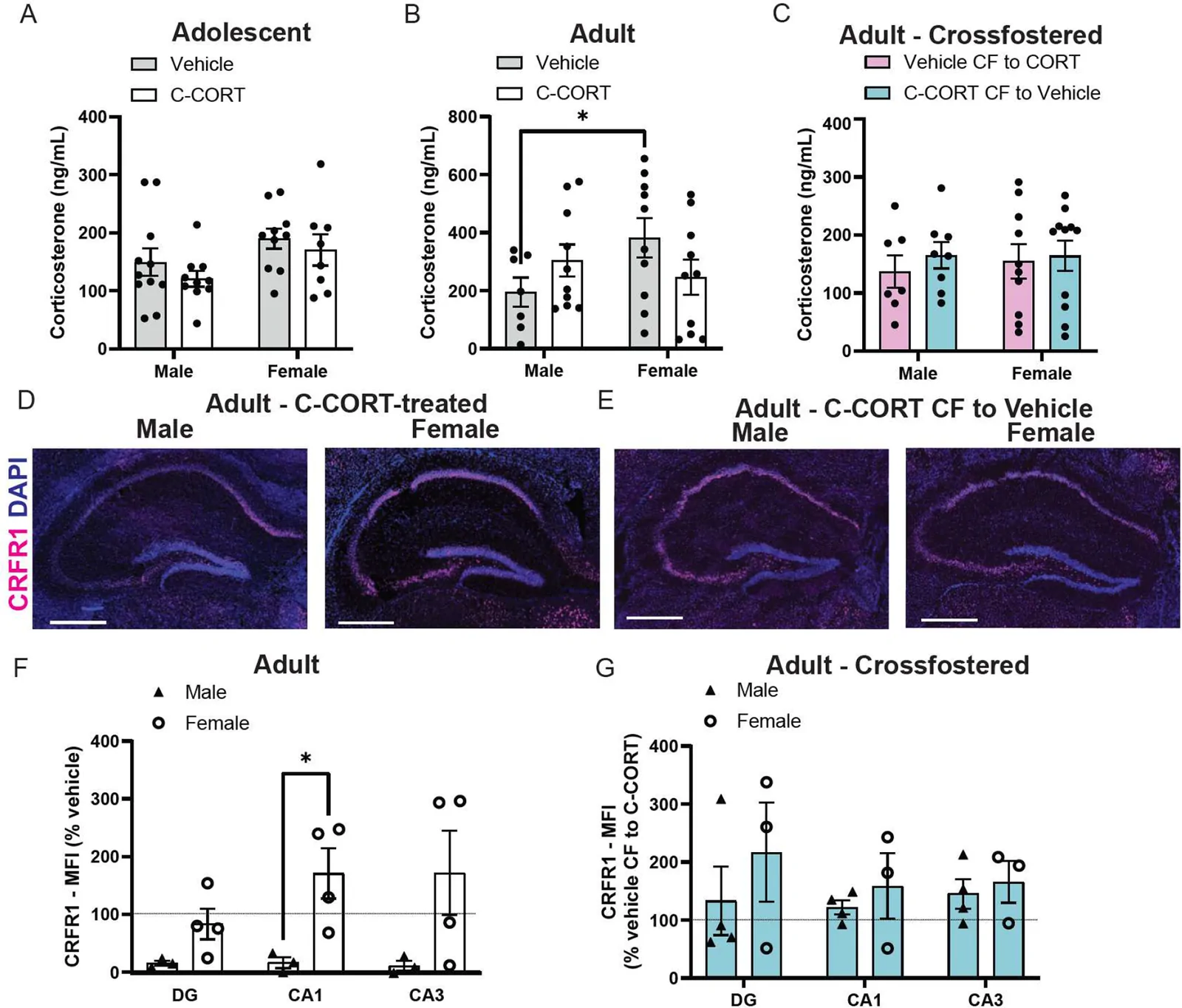
Cross-fostering offspring with prenatal C-CORT treatment normalizes sex-differences in circulating corticosterone and hippocampal CRFR1 expression. A. Circulating corticosterone in adolescent offspring is comparable among groups. B. Circulating corticosterone in adults is increased in female offspring with prenatal vehicle treatment (Two-way ANOVA main effect of sex × treatment interaction F_1,33_=4.005 p=0.0536, Fisher’s post-hoc test significant male vs. female vehicle-treated offspring *p=0.0470). C. Cross-fostered adult offspring circulating corticosterone levels are comparable among groups. Representative fluorescence in situ hybridization images of hippocampal CRFR1 expression (magenta) in D. Adult C-CORT-treated offspring male (left) and female (right) and E. Adult C-CORT cross-fostered to vehicle offspring male (left) and female (right) (DAPI, blue; scale bar 500 μm). F. Hippocampal CRFR1 expression is increased in females with prenatal C-CORT treatment (Two-way ANOVA main effect of sex F_1,5_=9.450 *p=0.0277, Tukey’s post-hoc test significant between male and female prenatal C-CORT-treated adult offspring *p=0.0353). G. Hippocampal CRFR1 expression is comparable among groups in cross-fostered adult offspring.

## Data Availability

With final revision, we will supply a supplemental excel file containing all the source data. Our analysis code is available at github.com/babdelme/AutryLab.

## References

[1] Burns ER, Farr SL, Howards PP, Centers for Disease C, Prevention. Stressful life events experienced by women in the year before their infants' births--United States, 2000–2010. MMWR Morb Mortal Wkly Rep. 2015;64(9):247–51. PMC5779604 25763877

[2] Lima SAM, El Dib RP, Rodrigues MRK, Ferraz GAR, Molina AC, Neto CAP, Is the risk of low birth weight or preterm labor greater when maternal stress is experienced during pregnancy? A systematic review and meta-analysis of cohort studies. PLoS One. 2018;13(7):e0200594. doi:10.1371/journal.pone.0200594 PMC6061976 30048456

[3] Ding X, Liang M, Wu Y, Zhao T, Qu G, Zhang J, The impact of prenatal stressful life events on adverse birth outcomes: A systematic review and meta-analysis. J Affect Disord. 2021;287:406–16. doi:10.1016/j.jad.2021.03.083 33838476

[4] Lautarescu A, Craig MC, Glover V. Prenatal stress: Effects on fetal and child brain development. Int Rev Neurobiol. 2020;150:17–40. doi:10.1016/bs.irn.2019.11.002 32204831

[5] Graignic-Philippe R, Dayan J, Chokron S, Jacquet AY, Tordjman S. Effects of prenatal stress on fetal and child development: a critical literature review. Neurosci Biobehav Rev. 2014;43:137–62. doi:10.1016/j.neubiorev.2014.03.022 24747487

[6] Bussières EL, Tarabulsy GM, Pearson J, Tessier R, Forest JC, Giguère Y. Maternal prenatal stress and infant birth weight and gestational age: A meta-analysis of prospective studies. Dev Rev. 2015;36:179–99. doi:10.1016/j.dr.2015.04.001

[7] Eberle C, Fasig T, Bruseke F, Stichling S. Impact of maternal prenatal stress by glucocorticoids on metabolic and cardiovascular outcomes in their offspring: A systematic scoping review. PLoS One. 2021;16(1):e0245386. doi:10.1371/journal.pone.0245386 PMC7822275 33481865

[8] Yang F, Janszky I, Roos N, Li J, Laszlo KD. Prenatal Exposure to Severe Stress and Risks of Ischemic Heart Disease and Stroke in Offspring. JAMA Netw Open. 2023;6(12):e2349463. doi:10.1001/jamanetworkopen.2023.49463 PMC10753395 38150252

[9] Sandman CA, Davis EP, Buss C, Glynn LM. Prenatal programming of human neurological function. Int J Pept. 2011;2011:837596. doi:10.1155/2011/837596 PMC3133795 21760821

[10] Talge NM, Neal C, Glover V, Early Stress TR, Prevention Science Network F, Neonatal Experience on C, Antenatal maternal stress and long-term effects on child neurodevelopment: how and why? J Child Psychol Psychiatry. 2007;48(3–4):245–61. doi:10.1111/j.1469-7610.2006.01714.x PMC11016282 17355398

[11] van den Bergh BRH, Dahnke R, Mennes M. Prenatal stress and the developing brain: Risks for neurodevelopmental disorders. Dev Psychopathol. 2018;30(3):743–62. doi:10.1017/S0954579418000342 30068407

[12] Wu Y, De Asis-Cruz J, Limperopoulos C. Brain structural and functional outcomes in the offspring of women experiencing psychological distress during pregnancy. Mol Psychiatry. 2024;29(7):2223–40. doi:10.1038/s41380-024-02449-0 PMC11408260 38418579

[13] Manzari N, Matvienko-Sikar K, Baldoni F, O'Keeffe GW, Khashan AS. Prenatal maternal stress and risk of neurodevelopmental disorders in the offspring: a systematic review and meta-analysis. Soc Psychiatry Psychiatr Epidemiol. 2019;54(11):1299–309. doi:10.1007/s00127-019-01745-3 31324962

[14] Weinstock M. The long-term behavioural consequences of prenatal stress. Neurosci Biobehav Rev. 2008;32(6):1073–86. doi:10.1016/j.neubiorev.2008.03.002 18423592

[15] Bock J, Wainstock T, Braun K, Segal M. Stress In Utero: Prenatal Programming of Brain Plasticity and Cognition. Biol Psychiatry. 2015;78(5):315–26. doi:10.1016/j.biopsych.2015.02.036 25863359

[16] McGowan PO, Matthews SG. Prenatal Stress, Glucocorticoids, and Developmental Programming of the Stress Response. Endocrinology. 2018;159(1):69–82. doi:10.1210/en.2017-00896 29136116

[17] Veru F, Laplante DP, Luheshi G, King S. Prenatal maternal stress exposure and immune function in the offspring. Stress. 2014;17(2):133–48. doi:10.3109/10253890.2013.876404 24417382

[18] Burgueno AL, Juarez YR, Genaro AM, Tellechea ML. Prenatal stress and later metabolic consequences: Systematic review and meta-analysis in rodents. Psychoneuroendocrinology. 2020;113:104560. doi:10.1016/j.psyneuen.2019.104560 31884321

[19] Bronson SL, Bale TL. The Placenta as a Mediator of Stress Effects on Neurodevelopmental Reprogramming. Neuropsychopharmacology. 2016;41(1):207–18. doi:10.1038/npp.2015.231 PMC4677129 26250599

[20] Lucas A. Programming by Early Nutrition in Man. Ciba Foundation Symposium 156 - The Childhood Environment and Adult Disease. p. 38–55. 1855415

[21] Sze Y, Brunton PJ. How is prenatal stress transmitted from the mother to the fetus? J Exp Biol. 2024;227(Suppl_1). doi:10.1242/jeb.246073 38449331

[22] Brunton PJ, Russell JA, Douglas AJ. Adaptive responses of the maternal hypothalamic-pituitary-adrenal axis during pregnancy and lactation. J Neuroendocrinol. 2008;20(6):764–76. doi:10.1111/j.1365-2826.2008.01735.x 18601699

[23] Krontira AC, Cruceanu C, Binder EB. Glucocorticoids as Mediators of Adverse Outcomes of Prenatal Stress. Trends Neurosci. 2020;43(6):394–405. doi:10.1016/j.tins.2020.03.008 32459992

[24] Cottrell EC, Seckl JR, Holmes MC, Wyrwoll CS. Foetal and placental 11beta-HSD2: a hub for developmental programming. Acta Physiol (Oxf). 2014;210(2):288–95. doi:10.1111/apha.12187 24330050

[25] Jensen Pena C, Monk C, Champagne FA. Epigenetic effects of prenatal stress on 11beta-hydroxysteroid dehydrogenase-2 in the placenta and fetal brain. PLoS One. 2012;7(6):e39791. doi:10.1371/journal.pone.0039791 PMC3383683 22761903

[26] Holmes MC, Abrahamsen CT, French KL, Paterson JM, Mullins JJ, Seckl JR. The mother or the fetus? 11beta-hydroxysteroid dehydrogenase type 2 null mice provide evidence for direct fetal programming of behavior by endogenous glucocorticoids. J Neurosci. 2006;26(14):3840–4. doi:10.1523/JNEUROSCI.4464-05.2006 PMC6445356 16597738

[27] Vaughan OR, Sferruzzi-Perri AN, Fowden AL. Maternal corticosterone regulates nutrient allocation to fetal growth in mice. J Physiol. 2012;590(21):5529–40. doi:10.1113/jphysiol.2012.239426 PMC3515836 22930269

[28] Leuner B, Fredericks PJ, Nealer C, Albin-Brooks C. Chronic gestational stress leads to depressive-like behavior and compromises medial prefrontal cortex structure and function during the postpartum period. PLoS One. 2014;9(3):e89912. doi:10.1371/journal.pone.0089912 PMC3940672 24594708

[29] Zoubovsky SP, Hoseus S, Tumukuntala S, Schulkin JO, Williams MT, Vorhees CV, Chronic psychosocial stress during pregnancy affects maternal behavior and neuroendocrine function and modulates hypothalamic CRH and nuclear steroid receptor expression. Transl Psychiatry. 2020;10(1):6. doi:10.1038/s41398-020-0704-2 PMC7026416 32066677

[30] Jafari Z, Mehla J, Afrashteh N, Kolb BE, Mohajerani MH. Corticosterone response to gestational stress and postpartum memory function in mice. PLoS One. 2017;12(7):e0180306. doi:10.1371/journal.pone.0180306 PMC5503237 28692696

[31] Bashiri H, Houwing DJ, Homberg JR, Salari AA. The combination of fluoxetine and environmental enrichment reduces postpartum stress-related behaviors through the oxytocinergic system and HPA axis in mice. Sci Rep. 2021;11(1):8518. doi:10.1038/s41598-021-87800-z PMC8055994 33875712

[32] Haim A, Albin-Brooks C, Brothers H, Breach M, Leuner B. Gestational stress disrupts dopamine and oxytocin signaling in the postpartum reward system of rats: implications for mood, motivation and mothering. Sci Rep. 2025;15(1):1450. doi:10.1038/s41598-024-84043-6 PMC11718260 39789137

[33] Champagne FA, Meaney MJ. Stress during gestation alters postpartum maternal care and the development of the offspring in a rodent model. Biol Psychiatry. 2006;59(12):1227–35. doi:10.1016/j.biopsych.2005.10.016 16457784

[34] Rivero-Quiles C, Abdelmesih B, Kong A, Khan U, Kislin M, Autry AE. Appetitive maternal behavior in mice is resistant to perinatal chronic variable stress and perinatal chronic corticosterone treatment. Horm Behav. 2026;177:105855. doi:10.1016/j.yhbeh.2025.105855 PMC12742011 41253068

[35] Gotz M, Huttner WB. The cell biology of neurogenesis. Nat Rev Mol Cell Biol. 2005;6(10):777–88. doi:10.1038/nrm1739 16314867

[36] Molyneaux BJ, Arlotta P, Menezes JR, Macklis JD. Neuronal subtype specification in the cerebral cortex. Nat Rev Neurosci. 2007;8(6):427–37. doi:10.1038/nrn2151 17514196

[37] Bond AM, Berg DA, Lee S, Garcia-Epelboim AS, Adusumilli VS, Ming GL, Differential Timing and Coordination of Neurogenesis and Astrogenesis in Developing Mouse Hippocampal Subregions. Brain Sci. 2020;10(12). doi:10.3390/brainsci10120909 PMC7760658 33255945

[38] Hayashi K, Kubo K, Kitazawa A, Nakajima K. Cellular dynamics of neuronal migration in the hippocampus. Front Neurosci. 2015;9:135. doi:10.3389/fnins.2015.00135 PMC4408843 25964735

[39] Wu Z, Autry AE, Bergan JF, Watabe-Uchida M, Dulac CG. Galanin neurons in the medial preoptic area govern parental behaviour. Nature. 2014;509(7500):325–30. doi:10.1038/nature13307 PMC4105201 24828191

[40] Abdelmesih B, Anderson R, Bambah-Mukku D, Carta I, Autry AE. Urocortin-3 neurons in the perifornical area are critical mediators of chronic stress on female infant-directed behavior. Mol Psychiatry. 2023;28(1):483–96. doi:10.1038/s41380-022-01902-2 PMC9847478 36476733

[41] Autry AE, Wu Z, Kapoor V, Kohl J, Bambah-Mukku D, Rubinstein ND, Urocortin-3 neurons in the mouse perifornical area promote infant-directed neglect and aggression. Elife. 2021;10. doi:10.7554/eLife.64680 PMC8452308 34423776

[42] Kohl J, Babayan BM, Rubinstein ND, Autry AE, Marin-Rodriguez B, Kapoor V, Functional circuit architecture underlying parental behaviour. Nature. 2018;556(7701):326–31. doi:10.1038/s41586-018-0027-0 PMC5908752 29643503

[43] Autry AE, Adachi M, Cheng P, Monteggia LM. Gender-specific impact of brain-derived neurotrophic factor signaling on stress-induced depression-like behavior. Biol Psychiatry. 2009;66(1):84–90. doi:10.1016/j.biopsych.2009.02.007 PMC2734472 19358977

[44] Zou J, Wang W, Pan YW, Lu S, Xia Z. Methods to measure olfactory behavior in mice. Curr Protoc Toxicol. 2015;63:11 8 1–8 21. doi:10.1002/0471140856.tx1118s63 PMC4339283 25645244

[45] Walf AA, Frye CA. The use of the elevated plus maze as an assay of anxiety-related behavior in rodents. Nat Protoc. 2007;2(2):322–8. doi:10.1038/nprot.2007.44 PMC3623971 17406592

[46] Lueptow LM. Novel Object Recognition Test for the Investigation of Learning and Memory in Mice. J Vis Exp. 2017(126). doi:10.3791/55718 PMC5614391 28892027

[47] Heck AL, Handa RJ. Sex differences in the hypothalamic-pituitary-adrenal axis' response to stress: an important role for gonadal hormones. Neuropsychopharmacology. 2019;44(1):45–58. doi:10.1038/s41386-018-0167-9 PMC6235871 30111811

[48] Van der Mierden S, Leenaars CHC, Boyle EC, Ripoli FL, Gass P, Durst M, Measuring endogenous corticosterone in laboratory mice - a mapping review, meta-analysis, and open source database. ALTEX. 2021;38(1):111–22. doi:10.14573/altex.2004221 33086382

[49] Spinedi E, Chisari A, Pralong F, Gaillard RC. Sexual dimorphism in the mouse hypothalamic-pituitary-adrenal axis function after endotoxin and insulin stresses during development. Neuroimmunomodulation. 1997;4(2):77–83. doi:10.1159/000097324 9483198

[50] Tartt AN, Mariani MB, Hen R, Mann JJ, Boldrini M. Dysregulation of adult hippocampal neuroplasticity in major depression: pathogenesis and therapeutic implications. Mol Psychiatry. 2022;27(6):2689–99. doi:10.1038/s41380-022-01520-y PMC9167750 35354926

[51] Jones KL, Zhou M, Jhaveri DJ. Dissecting the role of adult hippocampal neurogenesis towards resilience versus susceptibility to stress-related mood disorders. NPJ Sci Learn. 2022;7(1):16. 10.1038/s41539-022-00133-y PMC9288448 35842419

[52] Anacker C, Hen R. Adult hippocampal neurogenesis and cognitive flexibility - linking memory and mood. Nat Rev Neurosci. 2017;18(6):335–46. doi:10.1038/nrn.2017.45 PMC6261347 28469276

[53] Bannerman DM, Sprengel R, Sanderson DJ, McHugh SB, Rawlins JN, Monyer H, Hippocampal synaptic plasticity, spatial memory and anxiety. Nat Rev Neurosci. 2014;15(3):181–92. doi:10.1038/nrn3677 24552786

[54] Plank AC, Frey S, Basedow LA, Solati J, Canneva F, von Horsten S, Prenatally traumatized mice reveal hippocampal methylation and expression changes of the stress-related genes Crhr1 and Fkbp5. Transl Psychiatry. 2021;11(1):183. doi:10.1038/s41398-021-01293-y PMC7988147 33758173

[55] Zheng Y, Zhang YM, Tang ZS, Du JK, Guo DW, Xu YJ, Spatial learning and memory deficits induced by prenatal glucocorticoid exposure depend on hippocampal CRHR1 and CXCL5 signaling in rats. J Neuroinflammation. 2021;18(1):85. doi:10.1186/s12974-021-02129-8 PMC8019183 33810797

[56] Tao L, Li XX, Tu XR, Liu R, Xu JW, Lv YL, Hippocampal Crhr1 conditional gene knockout ameliorated the depression-like behavior and pathological damage in male offspring mice caused by chronic stress during pregnancy. Behav Brain Res. 2024;472:115139. doi:10.1016/j.bbr.2024.115139 38969017

[57] Maras PM, Baram TZ. Sculpting the hippocampus from within: stress, spines, and CRH. Trends Neurosci. 2012;35(5):315–24. doi:10.1016/j.tins.2012.01.005 PMC3423222 22386641

[58] Meek LR, Dittel PL, Sheehan MC, Chan JY, Kjolhaug SR. Effects of stress during pregnancy on maternal behavior in mice. Physiol Behav. 2001;72(4):473–9. doi:10.1016/s0031-9384(00)00431-5 11282130

[59] Smith JW, Seckl JR, Evans AT, Costall B, Smythe JW. Gestational stress induces post-partum depression-like behaviour and alters maternal care in rats. Psychoneuroendocrinology. 2004;29(2):227–44. doi:10.1016/s0306-4530(03)00025-8 14604603

[60] Brummelte S, Galea LA. Chronic corticosterone during pregnancy and postpartum affects maternal care, cell proliferation and depressive-like behavior in the dam. Horm Behav. 2010;58(5):769–79. doi:10.1016/j.yhbeh.2010.07.012 20688070

[61] Scarborough J, Iachizzi M, Schalbetter SM, Muller FS, Weber-Stadlbauer U, Richetto J. Prenatal and postnatal influences on behavioral development in a mouse model of preconceptional stress. Neurobiol Stress. 2024;29:100614. doi:10.1016/j.ynstr.2024.100614 PMC10865047 38357099

[62] Zoubovsky SP, Williams MT, Hoseus S, Tumukuntala S, Riesenberg A, Schulkin J, Neurobehavioral abnormalities following prenatal psychosocial stress are differentially modulated by maternal environment. Transl Psychiatry. 2022;12(1):22. doi:10.1038/s41398-022-01785-5 PMC8764031 35039487

[63] Maccari S, Piazza PV, Kabbaj M, Barbazanges A, Simon H, Le Moal M. Adoption reverses the long-term impairment in glucocorticoid feedback induced by prenatal stress. J Neurosci. 1995;15(1 Pt 1):110–6. doi:10.1523/JNEUROSCI.15-01-00110.1995 PMC6578279 7823121

[64] Belnoue L, Malvaut S, Ladeveze E, Abrous DN, Koehl M. Plasticity in the olfactory bulb of the maternal mouse is prevented by gestational stress. Sci Rep. 2016;6:37615. doi:10.1038/srep37615 PMC5122868 27886228

[65] Cuffe JS, Turton EL, Akison LK, Bielefeldt-Ohmann H, Moritz KM. Prenatal corticosterone exposure programs sex-specific adrenal adaptations in mouse offspring. J Endocrinol. 2017;232(1):37–48. doi:10.1530/JOE-16-0417 27754933

[66] Smith AL, Paul E, McGee D, Sinniah R, Flom E, Jackson-Humbles D, Chronic, Elevated Maternal Corticosterone During Pregnancy in the Mouse Increases Allergic Airway Inflammation in Offspring. Front Immunol. 2019;10:3134. doi:10.3389/fimmu.2019.03134 PMC6985541 32038643

[67] O'Sullivan L, Cuffe JS, Koning A, Singh RR, Paravicini TM, Moritz KM. Excess prenatal corticosterone exposure results in albuminuria, sex-specific hypotension, and altered heart rate responses to restraint stress in aged adult mice. Am J Physiol Renal Physiol. 2015;308(10):F1065–73. doi:10.1152/ajprenal.00676.2014 25715988

[68] Cuffe JS, Burgess DJ, O'Sullivan L, Singh RR, Moritz KM. Maternal corticosterone exposure in the mouse programs sex-specific renal adaptations in the renin-angiotensin-aldosterone system in 6-month offspring. Physiol Rep. 2016;4(8). doi:10.14814/phy2.12754 PMC4848720 27122048

[69] Paul EN, Shubitidze S, Rahim R, Rucker I, Valin L, Apostle S, Exogenous corticosterone administration during pregnancy in mice alters placental and fetal thyroid hormone availability in females. Placenta. 2023;142:1–11. doi:10.1016/j.placenta.2023.08.005 PMC10763606 37579594

[70] Taniguchi L, Goodpaster CM, Chen Y, de Carvalho GB, Birnie MT, Paul A, Sex and Stress Govern the Function and Innervation of a Basolateral Amygdala to Nucleus Accumbens Corticotropin-Releasing Hormone/GABA-Expressing Projection. J Neurosci. 2026;46(2). doi:10.1523/JNEUROSCI.1239-25.2025 PMC12809659 41271439

[71] Boersma GJ, Lee RS, Cordner ZA, Ewald ER, Purcell RH, Moghadam AA, Prenatal stress decreases Bdnf expression and increases methylation of Bdnf exon IV in rats. Epigenetics. 2014;9(3):437–47. doi:10.4161/epi.27558 PMC4053462 24365909

[72] Zheng Y, Fan W, Zhang X, Dong E. Gestational stress induces depressive-like and anxiety-like phenotypes through epigenetic regulation of BDNF expression in offspring hippocampus. Epigenetics. 2016;11(2):150–62. doi:10.1080/15592294.2016.1146850 PMC4846107 26890656

[73] Benoit JD, Rakic P, Frick KM. Prenatal stress induces spatial memory deficits and epigenetic changes in the hippocampus indicative of heterochromatin formation and reduced gene expression. Behav Brain Res. 2015;281:1–8. doi:10.1016/j.bbr.2014.12.001 PMC4305490 25496779

[74] Liu MY, Wei LL, Zhu XH, Ding HC, Liu XH, Li H, Prenatal stress modulates HPA axis homeostasis of offspring through dentate TERT independently of glucocorticoids receptor. Mol Psychiatry. 2023;28(3):1383–95. doi:10.1038/s41380-022-01898-9 PMC10005958 36481932

[75] Marchisella F, Creutzberg KC, Begni V, Sanson A, Wearick-Silva LE, Tractenberg SG, Exposure to Prenatal Stress Is Associated With an Excitatory/Inhibitory Imbalance in Rat Prefrontal Cortex and Amygdala and an Increased Risk for Emotional Dysregulation. Front Cell Dev Biol. 2021;9:653384. doi:10.3389/fcell.2021.653384 PMC8204112 34141707

[76] Monteleone MC, Adrover E, Pallares ME, Antonelli MC, Frasch AC, Brocco MA. Prenatal stress changes the glycoprotein GPM6A gene expression and induces epigenetic changes in rat offspring brain. Epigenetics. 2014;9(1):152–60. doi:10.4161/epi.25925 PMC3928178 23959066

