# Supplementary material for: Chronic prenatal corticosterone exposure alters postpartum maternal behavior and shapes sex-specific offspring outcomes
Source: Res Sq. 2026 Aug 14:rs.3.rs-10607522. Preprint. [Version 1] doi: 10.21203/rs.3.rs-10607522/v1 (PMC13640914; doi:10.21203/rs.3.rs-10607522/v1)
Supplement: Supplementary file 1 [file NIHPPRS10607522V1-supplement-1.pdf]

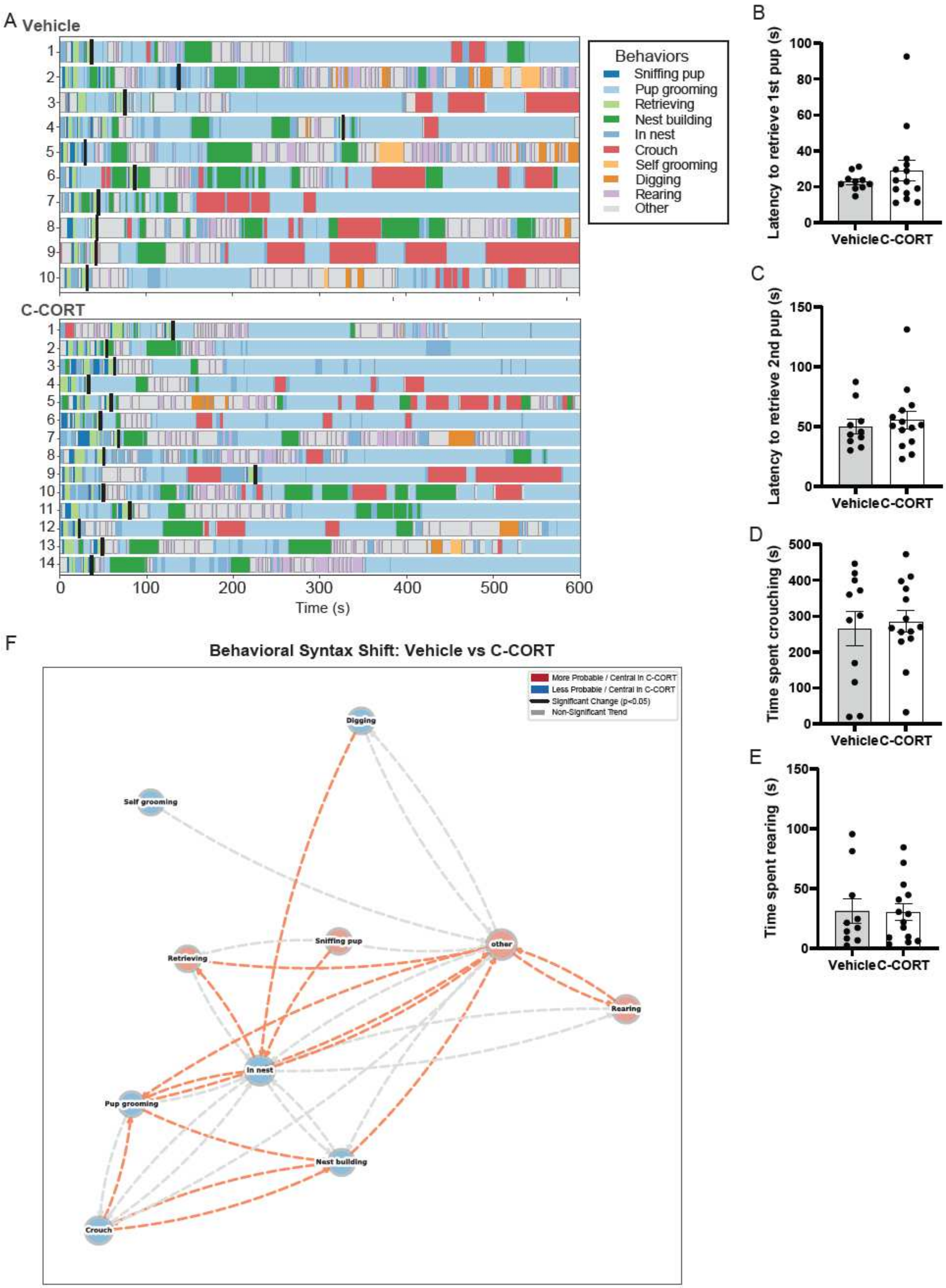

A
Vehicle
C-CORT
Behaviors
Sniffing pup
Pup grooming
Retrieving
Nest building
In nest
Crouch
Self grooming
Digging
Rearing
Other
Time (s)
0
100
200
300
400
500
600
B
Latency to retrieve 1st pup (s)
Vehicle
C-CORT
C
Latency to retrieve 2nd pup (s)
D
Time spent crouching (s)
E
Time spent rearing (s)
F
Behavioral Syntax Shift: Vehicle vs C-CORT
More Probable / Central in C-CORT
Less Probable / Central in C-CORT
Significant Change (p<0.05)
Non-Significant Trend
Digging
Self grooming
Retrieving
Sniffing pup
other
Rearing
In nest
Pup grooming
Nest building
Crouch

**Supplemental Figure 1. Additional maternal behavior measures.** A. Ethograms depicting pup-directed behavior across the 10-minute trial are shown for (top) Vehicle- and (bottom) C-CORT-treated dams (each row is one mouse, black bar indicates $2^{nd}$ pup retrieval). Latency to retrieve the B. first or C. second pup is not different between groups. We observed no group differences in D. crouching or E. rearing. F. Behavioral syntax shift comparing vehicle- and C-CORT-treated dams reveals no significant changes among nodes.
